# *Gloeostereum cimri*, a novel shelf fungus isolated from a human pulmonary cyst

**DOI:** 10.1080/22221751.2020.1769499

**Published:** 2020-05-31

**Authors:** Sarah A. Ahmed, Sybren de Hoog, Janet Kim, Jayne Crozier, Sarah E. Thomas, Benjamin Stielow, David A. Stevens

**Affiliations:** a Westerdijk Fungal Biodiversity Institute, Utrecht, The Netherlands; b Foundation Atlas of Clinical Fungi, Hilversum, The Netherlands; c Faculty of Medical Laboratory Sciences, University of Khartoum, Khartoum, Sudan; d Expertise Center in Mycology Radboud University Medical Center, Canisius Wilhelmina Hospital, Nijmegen, The Netherlands; e Santa Clara Valley Medical Center, San Jose, CA, USA; f CABI, Egham, UK; g Thermo Fisher Scientific, Landsmeer, The Netherlands; h California Institute for Medical Research, San Jose, CA, USA; i Division of Infectious Diseases and Geographic Medicine, Stanford University Medical School, Stanford, CA, USA

**Keywords:** Pulmonary infection, filamentous basidiomycetes, shelf fungi, crystals, *Gloeostereum*

## Abstract

Filamentous basidiomycetes are uncommon agents of human diseases, despite their ubiquitous presence in the environment. We present a case of symptomatic pulmonary infection in a 38-year-old male with cough and fever; a thin-walled cyst in the posterior left upper pulmonary lobe was revealed by radiography. A non-sporulating fungus was isolated from sputum and biopsy material from the cyst. ITS and LSU sequences placed the fungus phylogenetically in *Agaricales*, family *Cyphellaceae*, and identified it as a member of shelf fungi in *Gloeostereum,* but without identity to any known species. The new species is described as *Gloeostereum cimri*. The clinical strain showed high MIC to voriconazole (>8 µg/ml) but had low MIC to amphotericin B (0.5 µg/ml).

## Introduction

Filamentous basidiomycetes (mushrooms and shelf fungi) are important environmental organisms that play an essential role in the natural ecosystem. They can be found in a wide range of habitats as decomposers of plant litter and dead wood, or as symbionts of trees, whereas some are plant pathogens [1,2]. In the context of human use, mushrooms are a valuable source of food with many edible species, but some are very toxic causing poisoning and death to hundreds of people every year [1]. Mushrooms and shelf fungi are also medicinally important [3]. Rarely, filamentous basidiomycetes can be detrimental to human health in colonizing and infecting human lungs, particularly when the host is immunocompromised or otherwise has anatomically impaired lungs [4–6]; possibly such infections are underdiagnosed [4]. The great majority of these concern shelf fungi: the Atlas of Clinical Fungi [7] lists 13 species of shelf fungi *versus* three species of mushrooms. The filamentous basidiomycetes are encountered in a wide array of clinical manifestations, ranging from asymptomatic colonization and allergy to invasive systemic, eventually cerebral mycoses [4,7].

In general, infections by basidiomycetes are much less frequent than those by ascomycetes. The majority of clinically important basidiomycetes are yeasts or yeast-like genera, such as *Cryptococcus*, *Malassezia*, *Trichosporon*, and *Rhodotorula* [4,6,7], while also some infections owing to the large, plant-pathogenic groups of rust- and smut-fungi have been reported [7]. In the past, the non-sporulating, filamentous basidiomycetes from clinical materials were usually considered as unidentifiable contaminants [8]. In recent decades, with the increasing awareness owing to the development of molecular identification tools, a substantial number of clinical cases caused by filamentous basidiomycetes have been identified [9,10]. The most commonly reported species is *Schizophyllum commune*, which has been reported in approximately 114 cases, 77% of those involving the respiratory tract [4,11,12]. Exposure to the airborne spores that leads to allergic diatheses or colonization of the respiratory tract is one of the most common clinical manifestations of the diseases caused by filamentous basidiomycetes [4,13].

In the present communication, we report a case of symptomatic pulmonary infection caused by a novel basidiomycete species that will be described below as *Gloeostereum cimri*.

## Case report

**Figure 1.**
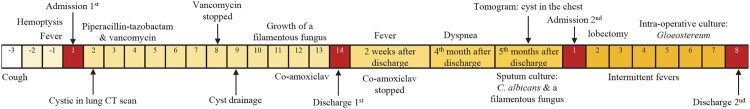
The chronological timeline of the patient's clinical course with essential findings and interventions. Numbers in blocks are days unless indicated otherwise.

A 38-year-old male presented with a cough for 3 days, hemoptysis, fevers, and chills for 1 day (Figure 1). He denied cough, fevers, night sweats, or weight loss prior to 3 days before admission. He had emigrated to the U.S.A. from Mexico seven years previously and had no foreign travel subsequently. In his previous life, he had worked as an automobile mechanic, in construction, and gardening. His past medical history indicated treatment for a lung infection, with an inability to work, 9 years previously, but he did not recall the diagnosis. During that time, he was treated concurrently with 3 different types of medications for 7 months but was never told that he had tuberculosis. He had no history of risk factors for an anaerobic lung abscess, such as aspiration, alcohol use, smoking or seizures.

On admission, his temperature was 39.4°C, leukocyte count was 6500/mm3, and HIV test was negative. Therapy with piperacillin-tazobactam and vancomycin was initiated and the patient remained hospitalized for 2 weeks. Computed tomography of the lung on hospital day 2 demonstrated an 8.2 × 5.5 cm cystic structure in the left upper lobe with an internal air-fluid level, fluid debris levels and a thick, irregular wall, consistent with a pulmonary abscess. There were surrounding areas of “ground-glass” opacities and consolidation in the left upper and lower lobes. Sputum cultures grew methicillin-susceptible *Staphylococcus aureus* and *Klebsiella oxytoca*, prompting discontinuation of vancomycin on hospital day 8. On hospital day 9, transthoracic left upper lobe pulmonary abscess drainage was performed, with an aspiration of 140 cc of dark green–black fluid. Cytologic examination of the aspirated fluid did not show malignant cells, but had abundant neutrophils, numerous erythrocytes, and necrotic debris. Smear and culture for mycobacteria, and anaerobic culture, were negative. Fungal culture yielded two colonies of a filamentous non-sporulating fungus. The therapy was then switched to oral amoxicillin-clavulanate. At discharge, his cough had improved and he denied dyspnea.

After discharge, he reported two weeks of mild intermittent dyspnea, but otherwise remained asymptomatic. The amoxicillin-clavulanate therapy was discontinued. However, serial chest radiographs demonstrated a persistent air-fluid level within a thin-walled cystic structure in the posterior left upper lobe. These radiographs were unchanged for four months. He reported two weeks of mild, intermittent dyspnea in the 4th-month post-discharge.

A chest tomogram performed 5 months post-discharge, to better characterize the disease, revealed a thin-walled cystic cavity in the left upper lobe apical posterior segment, measuring 5.0 × 4.6 cm. The fluid within the lesion was hyperdense, and there was a calcified component posteriorly. Cephalad to the lesion, there was an architectural distortion and bronchiectasis with adjacent scarring and likely adjacent cicatricial emphysema. The oblique fissure was displaced anteriorly, posterior to this region, secondary to the associated volume loss and/or scar. There was less consolidation in this area compared to prior tomograms.

A sputum bacterial culture was unremarkable, sputum smear for parasites was negative, as were antibody tests for *Histoplasma, Coccidioides* (IgG and IgM), and *Echinococcus*, and 2 blood cultures*.* A test for HIV was again negative. Sputum fungal culture grew *Candida albicans* and a filamentous fungus, morphologically similar to the previously isolated fungus. With a diagnosis of a persistent abscess, he proceeded to left upper lobectomy and extensive lysis of pleural adhesions. He was hospitalized 8 days, during which time he had intermittent fevers. Initially, there was a mild leukocytosis, which subsided. The intra-operative findings revealed an abscess that was located deep within the posterior aspect of the left upper lobe. The posterior fissure was completely fused and required extensive dissection. Intra-operative cultures were positive for a fungus, identified by sequencing as *Gloeostereum* sp. This was morphologically similar to the filamentous fungus that had been cultured on the previous occasions. Susceptibility testing of the fungus by macrobroth dilution indicated low MIC to amphotericin B (0.5 µg/ml), but high to voriconazole (MIC of >8 µg/ml) and fluconazole (MIC 16 µg/ml). *Candida albicans* was not cultured in the specimen, nor seen in the histopathology. No antifungal chemotherapy was given.

**Figure 2.**
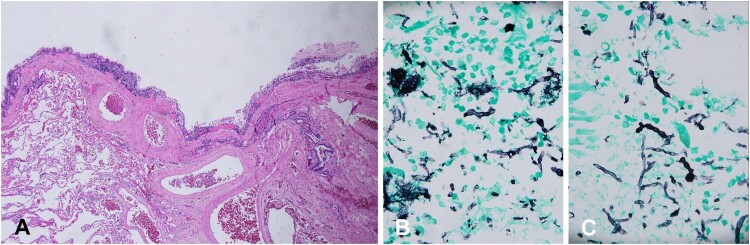
Histopathology section from the lung cyst showing (A) inflammation lining the cyst (Hematoxylin and Eosin). (B&C) Fungal hyphae (Grocott Methenamine Silver).

Histopathology of the resected specimen was interpreted as chronic inflammation in a bronchogenic cyst with a lining of ciliated pseudostratified columnar epithelium, some muscle and fibrous tissue, subepithelial chronic inflammation and pulmonary congestion (Figure 2). The cyst contents showed fungal hyphae (Figure 2). The fungus was sent to the Westerdijk Fungal Biodiversity Institute for definitive speciation and deposited there under number CBS 145006. The patient was seen in clinic 2 weeks post-discharge, at which time he denied fevers, chills. He remained well in subsequent visits.

## Material and methods

Strains isolated from sputum and lung biopsy from the patient were inoculated on Sabouraud's glucose agar (SGA, Oxoid, Basingstoke, England) and maintained at 24°C. The growth characteristics were studied on SGA, malt extract agar (MEA, Oxoid), oatmeal agar (OA; 2% oat meal), cherry decoction agar (CDA, homemade at Westerdijk Institute), X agar (XA, homemade at WI), Melin-Norkrans agar (MMN) [14] plates incubated for 2–4 weeks at 25°C and 30°C. To induce sporulation, MEA, MMN and OA cultured plates were incubated for 8 weeks in dark, in blue light (daylight), and under near-UV light. Slides were mounted in lactic acid and examined with Nikon Eclipse 80i microscope. For molecular identification, DNA was extracted from a fresh culture grown on MEA plates using the cetyltrimethyl ammonium bromide (CTAB) protocol of Möller et al. [15]. The rDNA internal transcribed spacer region (ITS), the large ribosomal subunit (LSU) and the small ribosomal subunit (SSU) were amplified using primers ITS1, ITS4, and LRoR, LR5, and NS1, NS24, respectively [16–18]. The above-mentioned primers were also used for sequencing of the corresponding genes except for SSU sequencing; for that purpose, additional primers were used including NS2, NS3, NS6, and NS7 [18]. Consensus sequences from forward and reverse primers were generated using Seqman assembly programme from Lasergene software package (DNASTAR, Madison, WI, U.S.A.). These sequences were compared to the GenBank sequence database using nucleotide BLAST tool. To further determine the phylogenetic affiliation of the strain, the ITS and D1-D2 domains of LSU were aligned with the close matches retrieved from GenBank. Phylogenetic analysis was performed using Bayesian inference (BI) approach in MrBayes v3.2.2 available at the CIPRES Science Gateway web server (www.phylo.org). The analysis was run for 20,000,000 generations with a sampling of one tree per 100 replicates, of them, 25% were discarded as a burn-in step. Strains that showed similarity in LSU phylogeny to our unknown isolate were subjected to molecular and phenotypic comparison using the same conditions as applied for the unknown isolate.

## Results

### Molecular identification

The BLAST comparison with GenBank sequence using partial SSU of the clinical isolate did not yield a match; the closest taxa differed by 15 bases from our isolate. With partial LSU, BLAST search revealed 99% similarity (803/806) to sequence accession number DQ327647 labelled *Homobasidiomycetes* sp. and 97% (799/809) to *Gloeostereum incarnatum* (AF141637). With rDNA ITS, similarity (99%) was found with the following unidentified strains: JQ919943 labelled as fungal sp., and KJ831953 labelled as *Agaricales* sp. Species that appeared among the best hits with both ITS and LSU, but at a rather low similarity, were *Chondrostereum purpureum* and *Gloeostereum incarnatum*.

**Figure 3.**
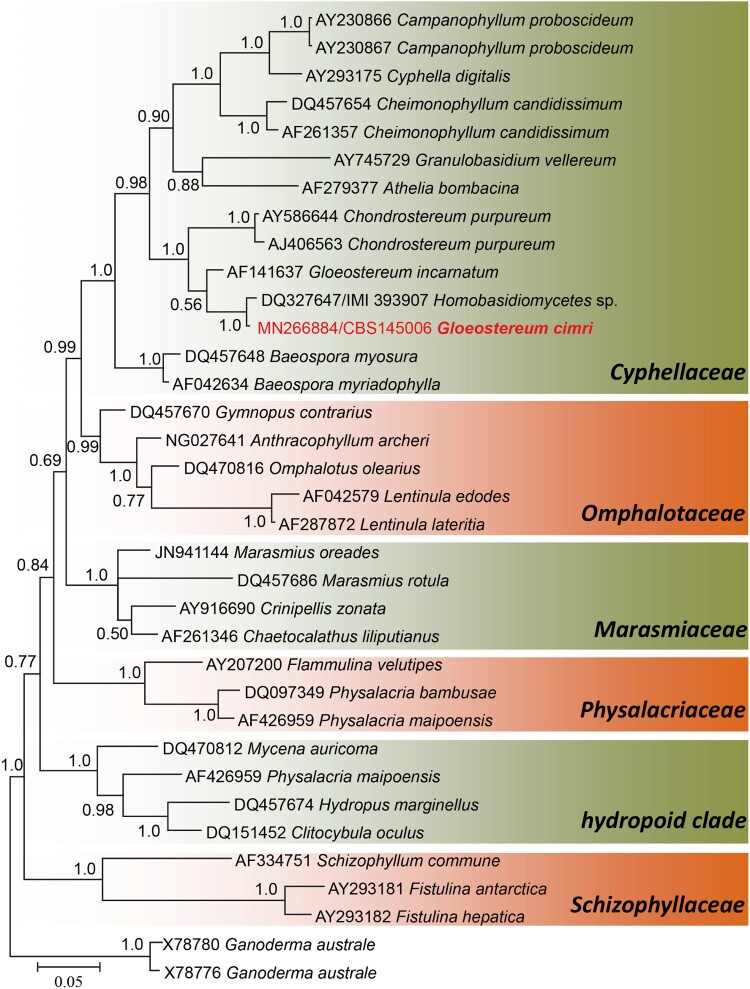
Phylogram generated from Bayesian analysis of LSU sequence of *Agaricales *families. Bayesian posterior probabilities (PP) are shown near the nodes. The clinical strain is in red bold text. The tree is rooted with *Ganoderma australe*.

In LSU phylogeny, 35 sequences were analyzed with a total of 741 alignment characters including gaps. In the LSU analysis, the clinical isolate clustered in the family *Cyphellaceae* of the order *Agaricales,* Basidiomycota. In *Cyphellaceae*, the isolate formed a well-supported clade with the isolate *Homobasidiomycetes* sp. (1.0 PP, Figure 3), which is in agreement with the BLAST comparison. The nearest neighbour to our clinical isolate, but without statistical support (0.56 PP), is *Gloeostereum incarnatum*. The sub-clade of *Cyphellaceae* that contains the clinical isolate and *G. incarnatum* formed a well-supported branch (1.0 PP) with *Chondrostereum purpureum* and was distant from other genera of the family. With both LSU phylogeny and BLAST comparison in GenBank, the clinical isolate appears to represent a novel taxon in the family *Cyphellaceae*.

**Figure 4.**
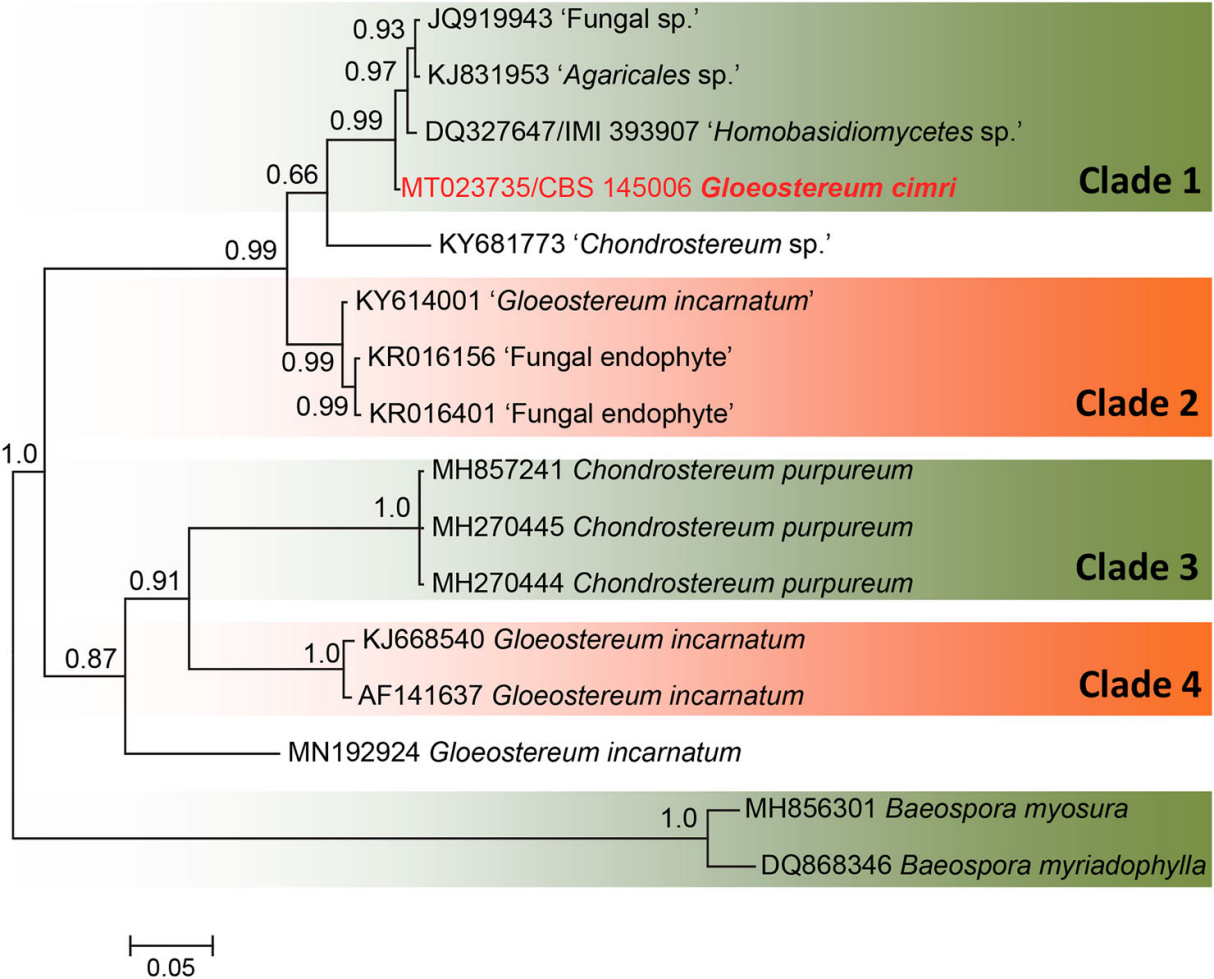
Phylogram generated from Bayesian analysis of ITS sequence of the clinical isolate and the close relatives. Bayesian posterior probabilities (PP) are shown near the nodes. The clinical strain is in red bold text. The tree is rooted with *Baeospora myosura* and *B. myriadophylla*.

In the ITS analysis, we included only the closest match to our unknown isolate, in addition to the found relatives in *Chondrostereum* and *Gloeostereum*. In this analysis, four clades were recognized (labelled clade 1–4), each representing a single phylogenetic species (Figure 4). Clade 1 (0.99 PP) contained our clinical isolate and three unidentified endophytes: one recovered from *Theobroma cacao* in Brazil [19] and two isolated from *Hevea* *brasiliensis* in Peru. Clade 2 (0.99 PP) contained three isolates, one of which was labelled *Gloeostereum incarnatum* and was isolated from wood in Thailand, while the other two were endophytes recovered from *Ficus pungens* in Papua New Guinea. Clade 3 contained the globally distributed plant pathogenic species *Chondrostereum purpureum*. The last clade (clade 4) contained additional *Gloeostereum incarnatum* isolates; one was a reference strain collected in 1990 and was used in the re-description of the species [20], while the other one was reported in 2015 from South Korea. Besides clade 2 and 4, another strain identified also as *G. incarnatum* was found in the basal lineage of the ITS tree, which adds to the heterogenous nature of the species. Although *Gloeostereum* is polyphyletic, it remains the oldest described genus in the clade containing our unknown isolate. Therefore, we propose *Gloeostereum cimri* as a new species for the clinical isolate, which is described below.

### Taxonomy

**Figure 5.**
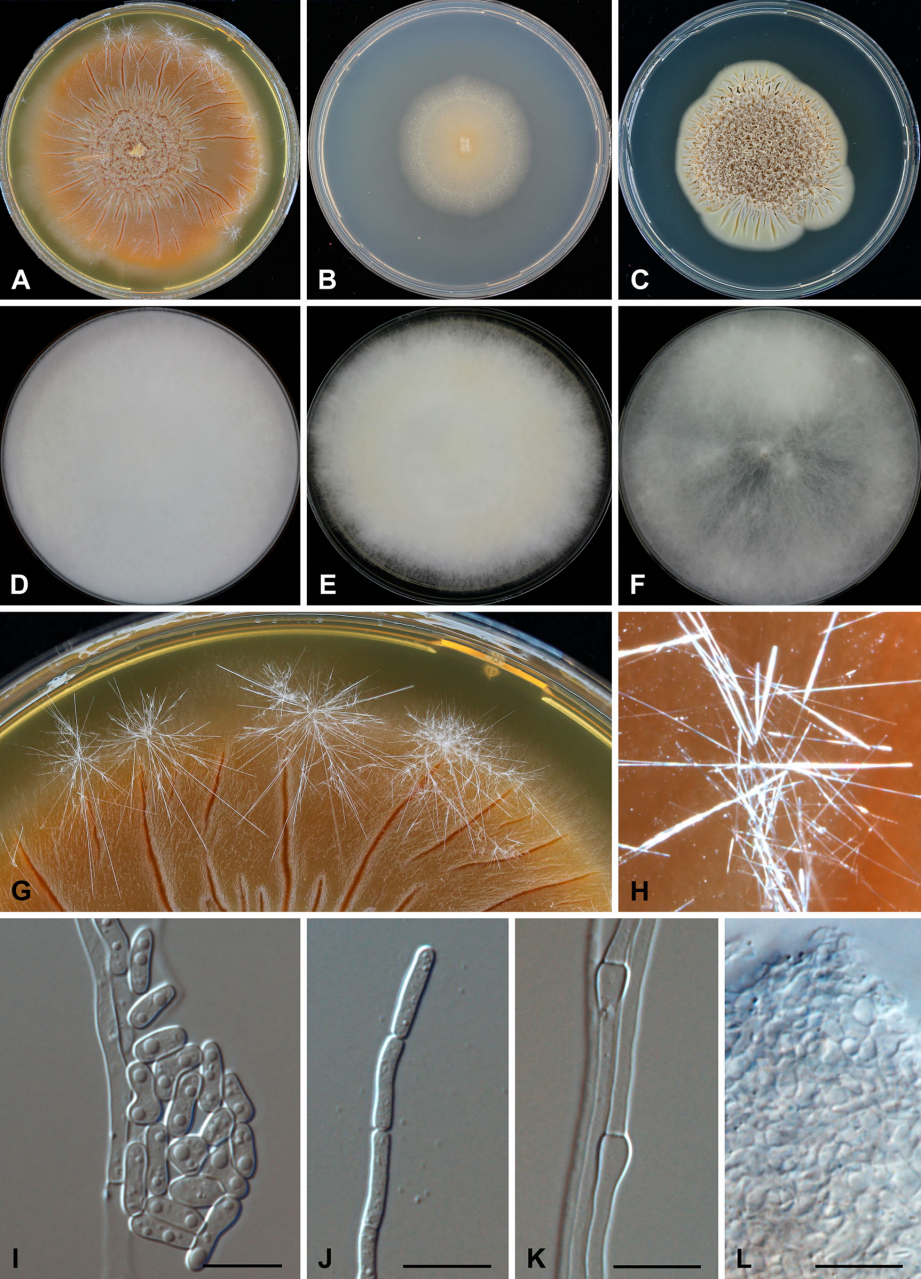
*Gloeostereum cimri*. Colonies after 4 weeks of incubation on (A) MEA, (B) MMN, (C) SABG. Colonies after 2 weeks of incubation on (D) MEA, (E) MMN, (F) SABG. (G & H) Crystals on the surface of MEA plates. (I & J) Arthroconidia. (K) Racquet hyphae. (L) interwoven hyphae. (A–C, G–L) CBS 145006. (D–F) IMI 393907. – Scale bars = 10 µm

*Gloeostereum cimri* S. A. Ahmed, D. A. Stevens & de Hoog, sp. nov. (Figure 5), MB 834197 *Etymology:* The species epithet refers to the first letters of California Institute for Medical Research, where the fungus was collected.

Colonies on MEA reaching 27 mm diam after two weeks of incubation at 30°C, while at 25°C the diameter was 15 mm, and at 37°C 12 mm. Colonies buff cream to pale brownish or dark honey-yellow, regular, thin, cerebriform at the centre and convoluted to flat with some aerial mycelium at the margin. Long and thin, glassy crystals were observed on the agar surface, mostly arranged in clusters. Colonies on OA creamy white, regular, flat and submerged, with some crystals on the agar surface. Colonies on MMN creamy whitish to pale yellow, regular, restricted, radiating with sectors and with a submerged margin, flat with few tufts of whitish aerial mycelium. Crystals were observed on the agar surface. Colonies on SGA creamy whitish on the surface with pale yellowish reverse, cerebriform flat or slightly heaped; crystals were observed in old cultures.

Hyphae thin, smooth, hyaline, septate and branched, 1.4‒3.2 µm wide (mean 2.29 ± 0.52). Some hyphae were swollen and intact, but no clump connections or fruiting bodies were observed. Terminally swollen hyphae (racquet hyphae) were present and some were locally coiled. Arthroconidia rare, mainly produced on MMN, mostly in the terminal part of the hyphae, thick-walled, rectangular, schizolytic, 2.1–3.9 µm wide (mean 2.79 ± 0.45).

*Type:* Living culture ex-type CBS 145006, San Jose, California, U.S.A., from a human lung biopsy specimen, D. A. Stevens & J. Kim. Additional environmental isolate examined: IMI 393907, Brazil, *Theobroma cacao*, J. Crozier & S. Thomas. This isolate was able to grow at 25 but not 37°C. Colonies were expanding with white, fluffy aerial mycelium; no crystals were observed. Arthroconidia were abundant, mostly arranged in clusters.

## Discussion

Patients with chronic lung diseases or anatomical lung damage are at risk of respiratory colonization by fungi [21]. *Aspergillus* and *Candida* are the most commonly reported fungi, not only as colonizers but also as allergic sensitizers, and invader in patients with immunological impairment [21–23]. In the case reported here, the patient presented with a symptomatic lung infection, and no clear genetic predisposition that might have contributed to his presentation was recognized. Although not specifically investigated, judging from his clinical history he appears not to have underlying cystic fibrosis or bronchiectasis. However, the patient reported treatment for a pulmonary disease 9 years prior to his presentation. He was treated for 7 months with 3 different medications. In the absence of medical records, disease and treatment remained unknown. Given the long treatment duration and the application of combination therapy, the patient probably had suffered from a chronic infectious disease, e.g. tuberculosis. Fungal colonization of pre-existing cavities and tubercular sequelae is quite common: of the 60 patients studied by Biswas et al. [24], tuberculosis was the predisposing factor in 26%. On the other hand, fungal colonization can also occur without apparent underlying disorders or risk factors, as proven by the example of a *Schizophyllum* bronchogenous cyst reported by Bulajic et al. [25].

In immunocompetent individuals, fungal pulmonary colonization generally remains asymptomatic, or with mild symptoms and self-recovery; persistence may occur in a few patients [26]. The patient reported by Bulajic et al. [25] was asymptomatic; the cyst was discovered coincidentally during a routine medical examination. The incubation period of* *fungal colonization varies depending on immunity and underlying condition of the patient [27]. For example, in tuberculosis patients, this period ranges from co-existence of the fungus with mycobacteria to years after infection [28]. In a case of pulmonary fungus ball described by Chawke et al., a patient had a history of cavitary pulmonary tuberculosis 10 years before his fungal presentation [29]. The evolution of the infection in our case is similar to the case described by Chawke et al. [29], in that the symptoms appeared 9 years after what might have been a tuberculosis infection.

Although *Aspergillus* species are considered the main cause of fungal respiratory infections and colonization, non-*Aspergillus* cases are increasingly recognized [8,30]. Among these are filamentous basidiomycetes (mushrooms and shelf fungi) which have previously only been known from their environmental habitat [4,8,30]. Shelf fungi, which usually produce leathery or hard shelf-like carpophores on decaying wood, seem to be much more frequent as pulmonary colonizers. Although the exact pathogenesis or virulence behind such colonization is not fully understood, these fungi are naturally adapted to decompose wood and other plant substrates by producing hydrolytic enzymes [31]. These enzymes can also be expressed during human infection to degrade the host cell and use it as a nutritious substrate for survival, and thus promoting the virulence of the fungus [31]. In addition, the ability to tolerate and grow at human body temperature as we have proved in our clinical isolate, is an important requirement for fungal pathogenicity [32].

Filamentous basidiomycetes that have been incriminated in fungal lung diseases are *Bjerkandera adusta, Emmia lacerata* (syn. *Ceriporia lacerata*), *Irpex lacteus, Perenniporia sp., Phanerochaete chrysosporium, Phanerochaete stereoides, Porostereum spadiceum, Schizophyllum commune, S. radiatum, and Trametes polyzona* [4–7,10,11,26,30–37]. In the clinical laboratory, isolates fail to produce identifiable or diagnostic structures [4], or fruitbodies remain amorphous [7]. Therefore, sequencing of ITS and D1/D2 domain of the LSU has been used to reveal isolates identity in clinical settings. Singh et al. sequenced 52 non-sporulating filamentous isolates from respiratory specimens, 50 of these were found to belong to basidiomycetes, and only two were non-basidiomycetous [5]. In another study, 31 of 50 randomly selected non-sporulating clinical strains were identified using ITS sequencing and found to be basidiomycetes; these strains were derived exclusively from respiratory specimens [38].

In the present case, a non-sporulating fungus was recovered from lung abscess and sputum of a patient with pulmonary symptoms. Only with ITS and LSU sequencing, the isolate could be classified with Basidiomycota, but without identity to any known species. Molecular identification of clinical basidiomycetes was initiated by de Hoog and Gerrits van den Ende [39], who applied ITS and partial SSU for identification of 11 filamentous strains. These authors were able to identify only six of the strains, while the remaining five were unidentifiable [39]. Romanelli et al. [12] applied sequence-based identification with ITS and partial LSU for 168 clinical basidiomycetes, of which only 82 could be identified down to genus level, and 48 to species level. The authors attributed the failure of identification to the few sequences of basidiomycetes deposited in GenBank [12]. Available sequences are increasing, such that Singh et al. in 2013 were able to identify 48 out of 52 basidiomycetes to species and one to genus level using ITS and partial LSU [5]. The remaining three isolates did not match any taxon and were reported as Basidiomycota sp. [5]. Such isolates, being not properly described and without a formal name, will remain poorly known even if they represent environmentally or clinically significant taxa. Therefore, for our clinical isolate, we decided to further investigate its phylogenetic and taxonomic position within Basidiomycota. The LSU placed the isolate in the order *Agaricales* and the family *Cyphellaceae*. This order also harbours the most clinically reported basidiomycete, *Schizophyllum commune*, although phylogenetically distant to our clinical isolate [11]. Within the *Cyphellaceae*, the isolate grouped with *Chondrostereum purpureum*, but phylogenetically closer to *Gloeostereum incarnatum*. *Chondrostereum purpureum* is a plant pathogenic species causing a fatal disease known as silver leaf of *Prunus*, while *Gloestereum incarnatum* is an edible and medicinal mushroom that is mainly found in Asia [20,40,41]

In the ITS phylogeny, analysed strains were grouped into four clades and two single- strain lineages. In addition to the undescribed environmental strain that was found to be close to our clinical isolate in the LSU tree [19], two more strains that also showed similarity to the clinical isolate were revealed with ITS (Clade 1, Figure 4). The ITS analyses clearly showed that a re-evaluation of strains affiliated to *G. incarnatum* and *C. purpureum* is required; *G. incarnatum* was found to be heterogeneous in the ITS analysis. *Gloeostereum* is a monotypic genus described for a shelf fungus that mainly grows on rotting wood of broad-leaved trees in eastern Asia. Judging from our molecular data, all taxa need revision, but our fragmentary representation of extant environmental diversity does not allow additional taxonomic changes. Therefore, based on current knowledge, our clinical isolate is described here as *Gloeostereum cimri*.

The genus *Gloeostereum* was described in 1933 and characterized by 2.5–15 cm wide, fan-shaped, sessile consoles arranged in tiers [20,42]. Because our clinical isolate remained without fruitbodies to be compared with *G. incarnatum*, we examined an environmental isolate (IMI 393907) for basidiocarp formation using different incubation conditions and growth substrates. The isolate only showed arthroconidia, similar to that formed by the clinical isolate. Arthroconidia are quite common in cultures of filamentous basidiomycetes [9]. *Gloeostereum cimri* is the first species not only in *Gloeostereum*, but also in *Cyphellaceae* to be reported from a clinical case. In contrast, *G. incarnatum* is an edible mushroom with medicinal activity and has been confirmed to produce antimicrobial compounds [43]. The compounds incarnatins A−E, incarnolactones A−C, incarnate methyl ester, incarnetic acid, and incarnatenin showed antimalarial, antituberculosis, and anti-*Bacillus* activity [44]. The species is geographically confined to China, Japan, Siberia, and Taiwan [20,45]. Our patient has not travelled to Asia and has only been in the Americas. It may be noted that the environmental isolates of *G. cimri* originate from Brazil and Peru, suggesting that *G. cimri* is a shelf fungus on the American continent [19,46].

To date, no interpretive breakpoints for *in vitro* antifungal susceptibility of basidiomycetes are available [4]. Our clinical isolates showed low MIC to amphotericin B which might indicate its susceptibility to this antifungal, while high MICs were recorded for voriconazole and fluconazole. Although the antifungal susceptibility was performed, the patients was improved after the excision of the cyst and no antifungal therapy was required.
